# Electro-Therapeutics

**Published:** 1880-05

**Authors:** Edward C. Mann

**Affiliations:** Superintendent Sunnyside Medical Retreat for Nervous Invalids; Member New York Neurological Society, Fort Washington; Member New York Medico-Legal Society, etc., New York City


(*To be continued.*)

---

ARTICLE II.

### ELECTRO-THERAPEUTICS.

BY EDWARD C. MANN, M. D.

Superintendent Sunnyside Medical Retreat for Nervous Invalids; Member New York Neurological Society, Fort Washington; Member New York Medico-Legal Society, etc., New York City.

" Hic illius arma,

Hic currus fuit."

We are happily arrived at a period in the history of medicine when an apology for a dissertation on the subject of electro-therapeutics is no longer needed, it having established for itself a reputation founded upon scientific experiment and experience. Although the history of electro-therapeutics dates far back to the earlier ages, yet the time from which anything of importance has been accomplished may be regarded to date from the researches of Duchenne, of France, in 1847, and Remak, of Berlin, in 1855, the former employing the faradic current and making the applications to the muscles, the latter employing the galvanic current and acting on the motor nerves.

Remak not only introduced the galvanic current, but was also the first discoverer of its catalytic action. He was the first to experiment scientifically on the galvanization of the sympathetic, brain and spinal cord, and in these researches added greatly to the science of electro-therapeutics. Since the time of which we have spoken the attention of many scientific men has been turned in this direction, and to-day many of our ablest medical men are engaged in investigations on this subject, and brilliant results have been, and are at this time being accomplished.

It is not our intention to attempt to present an exhaustive essay upon electro-therapeutics, but passing rapidly over the past history and present record of the results which have thus far been accomplished, to dwell more at length upon two subjects which have engaged our attention, from their having been hitherto comparatively neglected, by electro-therapeutists, although from their importance they are entitled to especial attention at the hands of those who are interested in the subject of medical electricity. We refer—

*First*, to the electro-therapeutics of diseases of the skin, and

*Second*, to the electro-therapeutics of displacements of the uterus.

### THE ELECTRO-THERAPEUTICS OF DISEASES OF THE SKIN.

Very little attention has as yet been paid to the treatment of skin diseases by electricity, although, from the constitutional source and central origin of many of them, the best results are to be hoped for. The relation that exists between the sympathetic and vaso-motor nerves and many cutaneous lesions is accepted by many dermatologists. The numerous experiments in which electricity and other remedial agents applied to the central nervous system have been efficacious, without the application of any local curative agent, have clearly proved the existence of such a relation. Neumann gives it as his opinion that "there is no doubt but that a large proportion of cutaneous lesions depends upon disorders of the vaso-motor nerves, which cause certain derangements of the circulation in the arteries, veins and cutaneous glands." We find that diseases of certain organs give rise to, or are accompanied by, certain cutaneous lesions. Thus in diseases of the stomach, especially disturbances of digestion, we often see eczema and urticaria. Upon the application of electricity in such cases we have invariably found very marked tenderness of the solar plexus, and in proportion as the dyspepsia was relieved the abnormal sensitiveness to the electrical current has diminished, and the general nutrition has improved, owing to the action of the electricity on the sympathetic nerve. In renal and uterine disorders we often see pruritus, eczema and urticaria, and the application of both the galvanic and faradic currents to the sympathetic nerve, acting on the solar and hypogastric plexuses, has been found in some cases to relieve the eruption, although in some of these cases the relief was but temporary, as the cutaneous lesion probably depended upon an incurable condition of the kidneys and uterus. In the cutaneous lesions which are the sequelae of scarlatina, measles, etc., acting on the vaso-motor nerves, the use of electricity which shall affect these nerves is certainly a mode of treatment from which we may reasonably expect good results.

In the cases that have presented themselves to our notice the most prominent symptoms relating to the nervous system have been "neurasthenia" or nervous exhaustion, anaemia, hysteria, neuralgia and insomnia; and in proportion as the nervous symptoms yielded to treatment the cutaneous lesions have disappeared. In none of the diseases of the skin do we find a more intimate relation existing between the nervous system and the cutaneous lesion than in herpes. Bärensprung thinks the origin of zoster to be an inflammation of the sympathetic fibre of the small spinal ganglion, which inflammation is communicated to the skin, and explains the neuralgia so frequently accompanying it by the transmission of the irritation and reflex action from the ganglion upon the posterior root. It has also been observed in some cases that inflammation of the entire nerve tract had existed. In the treatment of this disease, central galvanization and galvanization of the sympathetic may open to the profession a means of cure which will, perhaps, be of some service. For the benefit of those who are not acquainted with the practical application of electricity we may explain that "central galvanization" signifies the most direct method of influencing the great nervous centres by the following method: Apply the negative pole to the superior part of the epigastric region so as to affect directly the solar plexus, and the positive pole to the crown of the head or cranial centre, which is a very important point for affecting the brain; and also over the sixth and seventh cervical vertebrae, or cilio-spinal centre, at which point, by moving the electrode on either side of the spine, we affect the cervical sympathetic nerve, and also the pneumogastric, phrenic, and laryngeal nerves and brachial plexuses. This point is the most important region of the body in the application of electricity, as we can influence more important nerve tracts than at any other place. In conclusion, the positive electrode may be passed down the entire length of the spine, from the first cervical to the second lumbar vertebra or cauda equina, at which latter point the sciatic nerves and their branches are strongly affected. We desire in this connection to express our firm belief in the efficacy of central galvanization in the treatment of diseases of the nervous system, believing that in no other way can we exert so important an alterative action upon the great nervous centres with such uniformly good results. We would by no means, however, depreciate the beneficial results accruing from the use of proper medication, but are in favor of a combination of the two forms of treatment. The late Prof. Felix von Niemeyer* in speaking of the difference between the constant and the induced current, says: "I am fully convinced that the introduction of the former into practice is one of the most valuable advances of modern times, and that *in the constant current we have a means more powerful than any other, of modifying the nutritive conditions of parts that are deeply situated.*"

But asking the indulgence of our readers for this digression, we return to the subject at hand. In many cases of prurigo we have traced the cause to the central nervous system, and Cazenave and Romberg both consider it a neurotic disease. In one case that came under our notice, that was relieved by the use of central galvanization, there was intense itching, but no complaint was made of any neuralgic pains, which was contrary to the rule laid down by German authorities, that if the prurigo is neurotic in origin, neuralgia and not itching will be present. The case referred to followed a severe attack of scarlatina. In another case which came on after long-continued mental excitement, both slight neuralgic pains, wandering in character, and severe itching existed. While our knowledge of the therapeutic uses of central galvanization is still in its infancy, extensive observations have been made, and cases have been reported by eminent authority upon skin diseases in which local galvanization has accomplished the best results in many cutaneous lesions. Conspicuous amongst these is the cure by local galvanization, by Dr. Henry G. Piffard, of a case of scleroderma, which is the more interesting and valuable as there are but about forty cases of this disease on record, and but very few other

*Text-book of Practical Medicine, by Dr. Felix von Niemeyer, Vol. II. page 290.

reports of cure. Neumann* reports an interesting case of scleroderma in the clinic of Dr. F. Fieber which resisted all ordinary local treatment, and was cured at length by local galvanization. We treated a case of Elephantiasis Arabum, syn. Bucnemia Tropica, Barbadoes leg, etc., for eight months by local galvanization with very marked benefit to the patient. As this disease is but rarely met with in this country, a passing notice of its pathology may not be uninteresting. According to Neumann,† the disease "consists essentially of an increase in substance of the skin and subcutaneous cellular tissue, whereby the circumference of the affected portion of the body is very considerably increased."

Tilbury Fox‡ considers it a hypertrophy of the cellular tissues caused by the effusion of a blastema in connection with a large number of molecules, granules, free nuclei and nucleated cells. The veins are distended and the lymphics are obliterated. The internal organs are often in a state of fatty degeneration. The primary seat of the disease he considers to be the blood, while locally the lymphatics are primarily affected.

The case of which we speak is that of an Englishman, Mr. P——, 50 years of age; family history good; occupation, office of a distillery. About two years ago he went to his place of business perfectly well, as he thought, and upon his arrival home at night he found that he could not remove the stocking of the left leg, owing to great swelling, which was oedematous in nature. A small point, which he described to have looked like a small blister, then appeared, and from this point exuded a watery serum, which discharge increased in quantity as the cellular tissue was infiltrated and as the leg increased in size. The leg continued to increase in size until July 1, 1872, at which time we first saw the patient. At that time the circumference of the calf of the left leg measured twenty-five inches. The right leg had also become affected and was also hypertrophied, although to a much less extent than the other. The left leg presented the appearance of an immense corrugated scab, not a trace of healthy epidermis being visible as far as the knee. Above that point the tissue was much hypertrophied, but normal in appearance, with the exception of a diffused redness. On the anterior aspect of the left foot was an ulcer of considerable size which was very painful. The right leg was the seat

* Hand-book of Skin Diseases, page 296. † Ibid. page 287.

‡ Skin Diseases, page 182.

of an erythematous redness with some exfoliation of the epidermis. There was an ulcer on the right foot also, near the ankle, which gave rise to the most excruciating pain, especially at night, depriving the patient of rest. No external applications made heretofore had afforded any relief, either to the hypertrophy or the pain of the ulcers. On the 12th of July, 1872, the following application was made:

A copper plate, to which was attached the negative pole of the battery, was put under the right foot, which was thoroughly moistened with warm water, and the weight of the body as far as practicable rested on this foot. The positive pole attached to a large sponge was then rubbed over the entire surface of the thigh and leg, special attention being given to the course of the nerves. Although a strong current was used it was not felt by the patient until the electrode was placed upon the comparatively healthy tissues above the knee, when it was felt strongly. This application was continued about an hour. The patient was not seen again until the 16th of September, 1872. The large scab or thickened epidermis had now begun to peel off in large scales, leaving the normal tissue visible beneath. The patient could also move his leg, which he could not do before the application of electricity. The leg now measured round the calf 21½ inches, being a reduction of three inches and a half in size in a month's time. The hypertrophy of the entire limb was found to have decreased in like proportion. The size of the ulcer remained the same. A second application was now made (September 16), a current from 16 cells of the battery being applied to both limbs in the same manner as before, with the exception of making a strong application by a sponge electrode to the surfaces of both the ulcers, and lasting about ten minutes for each one, the application giving considerable pain. The length of séance as before was an hour. September 20, reports that he has had no pain since the morning of the 13th inst., which is a great relief, as he has hardly had a good night's rest before for a year. The scab is exfoliating and the leg decreasing in size. October 18, the treatment by local galvanization has been continued since September with the most gratifying results, the applications being made twice a week and lasting for about three-quarters of an hour. The diameter of the calf of the left leg is reduced to 17 inches. The ulcer upon the right foot has filled up with healthy granulations and is nearly cured. The ulcer on the other foot has also improved very much, the effect of the galvanic current having been to stop the discharge of an offensive sanious pus. The fungous, bleeding granulations which rose above the surface have disappeared. and small, healthy granulations are beginning to appear. Appetite good, bowels regular. Temperature 98° and pulse 70. December 23, has had no application for a month until to-day. Application of fifteen minutes in length to each leg. Ten days ago the pains in the right foot which had been relieved so much a month previous, but which had since troubled him, ceased entirely. Both ulcers in good healthy condition, the one on the right foot being nearly well. The diameter of the left leg around the calf is a trifle less than 17 inches. Up to the present date (February) the condition of the patient remains about same. This is the only case of the kind that has been treated by local galvanization, and so far as the knowledge of the writer extends, the only case that has been markedly relieved by any treatment. The patient is a very fleshy man, and he thinks the normal diameter of his calf to be about 16 inches or thereabouts.

That eczema may be ranked in many cases with the neuroses, and cured by central galvanization, is, we think, demonstrated by the following interesting cases:

CASE 1.—Miss O'C——. Eczema following intermittent fever. Has had four succeeding attacks, which reduced her health very much, leaving her very weak and anaemic. A short time after the last attack the legs began to swell, the swelling being accompanied with erythematous redness and vesicular eruption, discharging a watery exudation which formed yellowish crusts. This eruption was accompanied by burning pain and a great deal of itching. The hands were swollen. The eruption also appeared upon the head and back. Four séances of central galvanization completely cured her, the length of the applications being about fifteen minutes and being made twice a week. Has at the present time a good appetite and is gaining flesh. No sign of the eruption.

CASE 2.—Chronic eczema of four years' standing. Mr. D——. Four years ago the eruption appeared on his legs. Grew steadily worse under local treatment, the eruption extending up the legs and to the back and arms. The use of arsenic caused the eruption to disappear for the period of eight months, at the end of which time it reappeared, and has since been confined to the legs and ankles, with a little on the hands. Intense itching was complained of. Three séances of central galvanization caused the itching to stop entirely, and at the last application the eruption was nearly all gone.

CASE 3.—Chronic eczema of two years' standing. Miss D. E. B. has been suffering for two years from a painful eczematous eruption, which was located on the palms of the hands and the soles of the feet, and on the right elbow. She complained of considerable pain in the left leg and ankle. Had sore-throat some time ago. Her hair fell out in large quantities two years ago. Reported that she had been treated for syphilis. Six séances of central galvanization resulted in the disappearance of the eruption. Can now walk any distance.

CASE 4.—Eczema capitis of over twenty years' standing. Mr. N., mechanic. This case is at the present time under treatment. Was sent by Dr. Piffard, surgeon to the New York Dispensary for Diseases of the Skin. Local applications having accomplished but transitory good effects, it was deemed desirable to try electricity. The patient accordingly presented himself last September for treatment, which has been continued ever since twice a week, the séances lasting about twenty minutes. The patient said that the eruption made its appearance when he was a boy at school and compelled him to discontinue his studies, and has covered his head to a more or less extent ever since. He had also a very severe dyspepsia, so that he was obliged to regulate his diet carefully and abstain from any exercise for some time after a meal. After a few applications the dyspepsia ceased to trouble him, and with the exception of three weeks, during which time the patient stayed away on account of sickness in his family, which, by reason of sleeplessness and nervous excitement, brought on an attack of dyspepsia, he has not suffered at all, to speak of, to the present time. The eczema of the scalp has improved very much, but is not entirely cured. His general health, however, is greatly improved, and he says he is better than he has been for years.

Marked relief has also followed the use of general faradization in two cases of skin diseases which seemed to depend upon uterine disorders which greatly reduced the general health and impaired the digestive functions. One of these cases occurred in a lady of about thirty-five years of age. A severe pruritus had existed for some months associated with dyspepsia. A few applications of general faradization restored the appetite and cured the dyspepsia and pruritus. After some time, however, the dyspepsia reappeared accompanied with a slight degree of pruritus, and it was not until the uterine trouble, which was prolapsus in the first degree, was cured that the pruritus entirely disappeared.

The other case referred to occurred in a young lady of twenty, who had suffered from amenorrhœa for some months. She complained of severe recurring attacks of urticaria with dyspepsia and nausea. Three applications of the induced current locally to the uterus and hypogastric region resulted in the reappearance of the menstrual flow, and a few applications of general faradization improved the general health very much, and the urticaria and dyspepsia disappeared. Up to the present time no complaint has been made of a recurrence of these symptoms.

(*To be continued.*)

